# Participatory Epidemiological Study of Endemic Bovine Diseases Among Gnyangatom and Dasenech Pastoralists, South Omo Zone, Ethiopia

**DOI:** 10.1155/vmi/2726225

**Published:** 2025-08-20

**Authors:** Yebelayhun Mulugeta, Debele Hordofa, Defar Elias

**Affiliations:** ^1^Department of Veterinary Medicine, Jinka University, Jinka, Southern Ethiopia, Ethiopia; ^2^Department of Biology, Jinka University, Jinka, Southern Ethiopia, Ethiopia

**Keywords:** Dasenech, endemic diseases, Gnyangatom, indigenous knowledge, participatory epidemiology

## Abstract

A community-based cross-sectional study was conducted, in which participatory appraisal methods were applied to validate Dasenech and Gnyangatom pastoralists' existing veterinary knowledge on endemic diseases and to determine their perception of rank, morbidity, and mortality of the disease. The participatory methods used were matrix scoring, proportional piling, pairwise ranking, and clinical observation. A total of 96 informants were included in the study to collect in-depth information. Ranking using proportional piling (*W* > 0.38;*p* > 0.05) and pairwise ranking (*p* > 0.05) based on the impact on livelihood revealed that anthrax, contagious bovine pleuropneumonia (CBPP), trypanosomiasis, pasteurellosis, and lumpy skin disease (LSD) were the five most important diseases prioritized in the study areas. The study also showed that these diseases' relative prevalence rate (participatory epidemiology (PE)-morbidity) was 18%, 16.5%, 6%, 8.5%, and 3.4%, respectively. The results also revealed that these diseases had a 14%, 10.88%, 5.58%, 5.3%, and 2% case fatality rate (PE-fatality). The analysis of matrix scoring indicated that pastoralists could associate the diseases with their clinical signs (*W* = 0.382; *p* < 0.05). Thus, the concordance between informant groups and participatory appraisal methods has proven that Dasenech and Gnyangatom pastoralists are knowledgeable about endemic bovine diseases. Hence, it is recommended that disease control intervention measures in the area should appreciate community involvement and consider these diseases.

## 1. Introduction

Livestock production is an integral part of the agricultural system in Ethiopia. Ethiopia has the largest livestock population in Africa, possessing more than 70 million cattle, 95.4 million small ruminants (42.9 million sheep and 52.5 million goats), 8.1 million camels and 13.33 million equines, and 57 million chickens [1].

This livestock sector has contributed a considerable portion to the economy of the country and is still promising to rally around the economic development of the country. Livestock production remains crucial and represents a major asset among resource-poor smallholder farmers by providing milk, meat, skin, manure, and traction force [1]. In Ethiopia, livestock production contributes 40% of the agricultural gross domestic product (GDP) and 20% of the total GDP without considering other contributions like the provision of traction power, organic fertilizers, and as a means of transport [2]. The contribution of livestock to the national economy, particularly foreign currency earnings, is through the exportation of live animals, meat and skin, and hides [3].

In Ethiopia, pastoralism is extensively practiced in almost two-thirds of the national land area. The sector is also important to the national economy, contributing 16% of total GDP, one-third of agricultural GDP, and 8% of export earnings. Improvements in the sector, therefore, have the potential to contribute significantly to national income and the welfare of many poor pastoral families [4].

Management of livestock such as cattle, goats, sheep, and camels is the primary livelihood of pastoral communities in the country. Livestock are critical to the well-being of pastoral households in terms of income, savings, food security, and employment. They rely heavily on dairy and livestock products as a livelihood system that provides for basic needs. The importance of livestock in these areas surpasses the mere fact of meeting basic needs, since they are traditionally seen as the basis of life, wealth, and social respect [4–6].

Though the livelihood of the pastoral community of Ethiopia is mainly dependent on livestock production, there has been a great coverage of pastoral areas with inadequate veterinary and health infrastructures and facilities (less supply of medical inputs/inadequate supply of treatment drugs), low number of health professionals, harsh environmental conditions, socioeconomic and cultural practices, traditional husbandry and poor management practices, mixing of wild animals with farm animals, and unrestricted movement [4, 6]. All these constraints, compounded with other factors, have exposed the pastoralist's cattle to different diseases.

Among these cattle diseases, endemic diseases of livestock are a major constraint to animal health, livestock production, and the economies of a pastoral community of Ethiopia by hundreds of millions of dollars. These diseases can reduce herd and flocks dramatically, which, in the case of pastoral people, is a major blow to food insecurity and the ability to survive. These diseases can erode the assets, thereby reducing the income of pastoralists, causing severe food shortages and access at the household level.

Control and surveillance have been progressively scaled back in many developing countries in favor of emerging, transboundary, and zoonotic diseases [7]. Even though any challenge facing pastoral livestock will directly affect the community, endemic disease management is left to livestock owners and private sector service providers. Cattle holders often fail to control endemic diseases in an economically optimal manner. The presence of such diseases is often unrecognized or considered “normal” and is thus fatalistically accepted by livestock holders.

However, there is a growing understanding that rural people are knowledgeable about the many subjects that touch their lives and that they possess a creative and analytical capacity that can greatly assist in improving agricultural practices [8]. Most livestock keepers know a lot about animal diseases and their different clinical presentations as they occur in the local area. Participatory epidemiology (PE) aims to explore this existing knowledge with communities and key informants to better understand the local disease situation and to enable disease prioritization. [9]. Because of these and other reasons, in pastoral areas, like the South Omo Zone of Ethiopia, PE approaches and methods are preferable to identify the range of diseases known to cattle owners and to indicate their status in the areas. Solutions developed through this approach would be action-oriented rather than data-driven, which is appropriate to pastoralists' technical level and culture, thus increasing their acceptability and sustainability [10, 11].

Mariner and Paskin [12] defined PE as “it is an emerging field that is based on the use of participatory techniques for the collection of qualitative epidemiological information contained within the community observations, existing veterinary knowledge and traditional oral history.”

Despite the importance of PE studies, there has been limited research in the pastoral areas of Ethiopia. It was this situation, along with aforementioned conditions, that gave us a background to design this study to make attempts to fill the gap and to assess the ethnoveterinary knowledge of Gnyangatom and Dasenech communities.

## 2. Materials and Methods

### 2.1. Description of Study Areas

**Figure 1:**
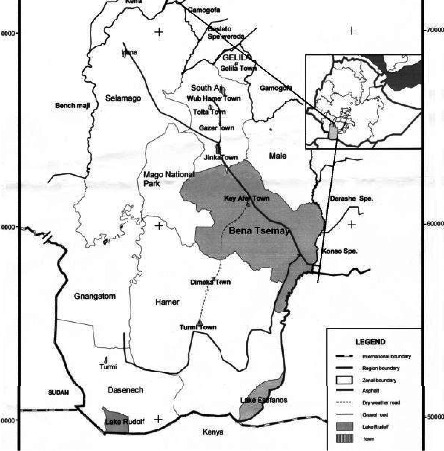
Map of the study areas (source: [13]).

The study was conducted in Gnyangatom and Dasenech districts of South Omo Zone, Ethiopia (Figure 1). The study areas were selected based on their potential for cattle population, close to the Mago National Park, and the existence of the problem.

Gnyangatom Woreda is one of the six districts in South Omo Zone with an area of 2652 sq. km. The area is situated at an astronomical location of 4.850–5.670°N and 35.750–36.230°E. The district is bordered in the North by the Bench–Maji zone and Selamago district, the Dasenech district in the South, the Hamar district in the East, and Kenya and South Sudan in the West. The agroecology of the district is tropical with an altitude that ranges between 300 and 450 m a.s.l. The mean annual temperature of the district ranges between 33°C and 42°C. The district has a rainfall pattern of bimodal type (Belg from March to May and Meher from August to October). The mean annual rainfall ranges from 350 to 500 mm. Livestock production is the dominant livelihood source in the district. It has an animal resource with an estimate of about 500,586 cattle, 154,385 goats, 126,940 sheep, 24,171 donkeys, and 6247 chickens (data from the Gnyangatom district Livestock and Fishery Development Office).

Dasenech district is found in South Omo Zone and bordered by Kenya in the South, Selamago district in the North, Gnyangatom in the West, and Hammer district in the East. It lay astronomically at 5°.14′N latitude, 36°.44′E longitude, and is 225 km from Jinka Town. The district covers a total area of 234,274 ha, with elevation ranging from 275 to 400 m. It has only one agroecological zone—Tropica. The mean annual rainfall of the district is about 350 mm. The annual mean temperature ranges from 30°C to 40°C. Livestock production is the main means of livelihood in the area. The most common livestock types are cattle, goats, sheep, donkey, and poultry, with a total population of 219,380, 206,185, 105,405, 14,394, and 5231, respectively. There are two rainy seasons: the main rainy season ranging from March through mid-April and the short rainy season from September through October (data from the Dasenech Woreda Livestock and Fishery Development Office).

### 2.2. Study Design

A community-based cross-sectional study was conducted to investigate the Gnyangatom and Dasenech communities' veterinary knowledge (experience) on endemic cattle diseases through the process of participatory disease investigation methods. The study population was key informants who were residents in the two districts with different sociodemographic characteristics. Besides this, the target population was interviewed with specific semiqualitative checklist questions related to animal disease mortality and morbidity knowledge.

### 2.3. Selection of Kebeles and Informants

Key informants were selected purposively based on the willingness of the livestock owners to participate in the study, livestock rearing experience, and their social value, which was essential to collect in-depth information on several issues. The optimum and manageable size of a household and key informants recommended in the participatory disease surveillance (PDS) interview are assumed to be 10–15 persons [12], and thus, 12 informants will be determined in each group. A total of 96 informants from randomly selected 4 kebeles (lower administrative units) of each district were included in the study.

### 2.4. Study Methods

Data were collected using the semistructured interview (SSI) and participatory techniques (tools) and principles. Participatory appraisal is largely based on qualitative techniques of gathering animal health information. Two important principles, which were designed to improve the quality and reliability of the information, triangulation and flexibility, were used. Triangulation is the method by which information is gained from several intentionally different perspectives. Flexibility is an essential principle where the techniques were not rigidly preplanned and executed without deviation but used unstructured questions that can be changed at any point during the investigation [14].

#### 2.4.1. SSIs

SSI was used extensively in connection with other PE tools such as clinical observation, matrix scoring, proportional piling, and pairwise ranking. Through these methods, we prioritized the major diseases of cattle in the study area, and using these data, we determined the morbidity and mortality of important endemic cattle diseases in the study area.

#### 2.4.2. Proportional Piling

**Figure 2:**
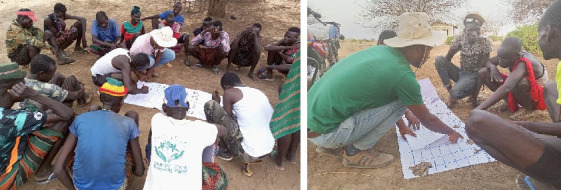
Proportional piling.

Proportional piling was employed to identify and rank the five most important cattle diseases and to estimate relative morbidity and mortality caused by these diseases during the past year (Figure 2). Before performing the proportional piling, informants were asked to classify the animals into different age groups. Again, informant groups were distributed a pile of 100 counters according to the impact each disease had on their livelihood to rank the five most important cattle diseases. Then, informant groups were asked to separate the proportion of a pile of 100 counters to show sick and healthy animals. This results in sick and healthy piles of counters. Next, informants were given the sick pile of counters for the five diseases and then separated into animals surviving and animals dying.

#### 2.4.3. Matrix Scoring

**Figure 3:**
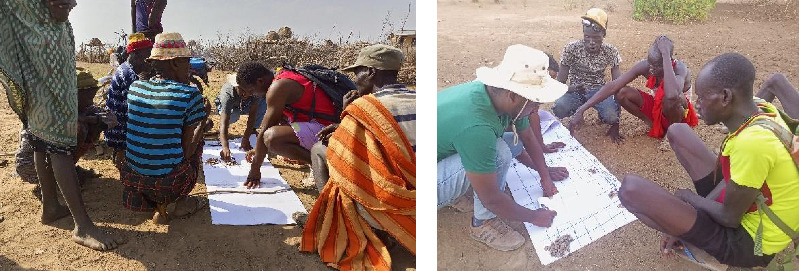
Matrix scoring.

Matrix scoring was used in community meetings to assess the ability of livestock keepers to clinically diagnose cattle diseases and to determine whether keepers associate the diseases with their clinical signs (Figure 3). The five most important diseases were placed along the *X*-axis of the matrix. The indicators (clinical signs) were illustrated along the *Y*-axis of the matrix. Informants were asked to use a total of 25 counters.

#### 2.4.4. Pairwise Ranking

**Figure 4:**
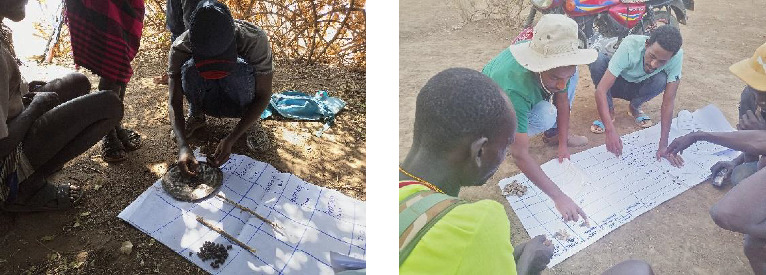
Pairwise ranking.

Pairwise ranking was used to understand the relative importance of diseases by comparing individual cattle diseases with each cattle disease one by one and through probing to understand the impact of different diseases (Figure 4). The names of cattle diseases were written vertically (*y*-axis) and horizontally (*x*-axis). For each pair of diseases (*x*, *y*), participants were asked which disease was more important.

### 2.5. Data Management and Analysis

The ordinal data obtained from the PE tools were stored in Microsoft Excel 2013 and later exported to SPSS Version 21 for analysis. Descriptive statistics (median, mode, range, percentages) were generated from the quantified participatory data for interpretation and comparison. To transform the qualitative local knowledge into semiquantitative data in the proportional piling exercise, the 100 counters (e.g., stones or beans) allocated to each disease within a group were counted and treated as a percentage of the total (100%). The number of counters the participants placed on the particular disease out of 100 total was recorded as a percentage of the perceived disease burden. Two nonparametric statistical tests were employed: Kendall's coefficient of concordance (*W*) and Friedman's rank test. Kendall's coefficient measured the agreement among informant groups based on data obtained from proportional piling and disease matrix scoring. Kendall's coefficient of concordance evaluates values ranging from 0 (no agreement) to 1 (complete agreement). Agreements can be categorized as weak (*W* < 0.26), moderate (*W* between 0.26 and 0.38), or good (*W* > 0.38). Friedman's rank test was used to assess the agreement among informant groups based on the data acquired from pairwise ranking.

## 3. Results

### 3.1. Major Livestock Species Kept

**Figure 5:**
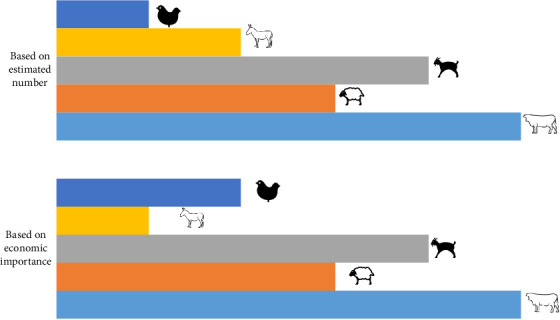
Rank of livestock based on population and importance.

Informants in selected kebeles of Gnyangatom and Dasenech ranked the livestock based on their perception of abundance and economic value for their livelihood (Table 1, Figure 5).

Taking a value represented by the greatest number of farmers (mode or the most frequently observed data) and cross-tabulating this with zonal livestock population data, the informants were ranked based on the highest population size as cattle, goats, sheep, chicken, and donkey and ranked based on the economic value as cattle, goats, sheep, donkey, and chicken (Table 1).

### 3.2. Common Diseases and Their Clinical Description

Endemic diseases within the knowledge scope of the informants were discussed through SSI. The respondents mentioned eleven (11) diseases that affect their cattle across the districts (Table 2). Types of diseases occurring in the area were listed with the local name (vernacular name), and the corresponding scientific name of these diseases was also given based on their clinical definitions (Table 2).

### 3.3. Proportional Piling

There was a significant good agreement (*W* > 0.38) between informant groups (*W* = 0.871). *p* value is greater than 0.05 (*p*=0.66). Hence, there is no significant difference between the districts/kebeles in ranking the diseases.

According to the informant's ranking in the current study, it showed that the five diseases in Dasenech district were anthrax, contagious bovine pleuropneumonia (CBPP), trypanosomiasis, pasteurellosis, and LSD; and CBPP, anthrax, trypanosomiasis, pasteurellosis, and foot and mouth disease (FMD) were in the Gnyangatom district. Having the overall median score of proportional piling exercise in the present study districts, the top five diseases were anthrax, CBPP, trypanosomiasis, pasteurellosis, and LSD (Table 3).

### 3.4. Pairwise Ranking

After proportional piling for frequency of occurrence, informants conducted pairwise ranking of the five selected diseases based mainly on morbidity and mortality rates, loss of production, and impact on the cattle keepers' livelihood (Table 4).

As indicated in Table 4, the mean ranks tell us which diseases were rated best versus least (the mean ranks tell us which disease was rated most favorably). Accordingly, the result of pairwise ranking of diseases in the present study revealed anthrax, CBPP, trypanosomiasis, pasteurellosis, and LSD as the five overall best-rated priority cattle diseases (most frequently occurring diseases) in terms of impact on livelihoods.

A Friedman test indicated that the *p* value is greater than 0.05 (*p*=0.112). Hence, there is no significant difference between the kebeles/districts. That is, the study districts/kebeles rated the relative importance of cattle diseases similarly.

### 3.5. Morbidity of the Five Diseases

**Figure 6:**
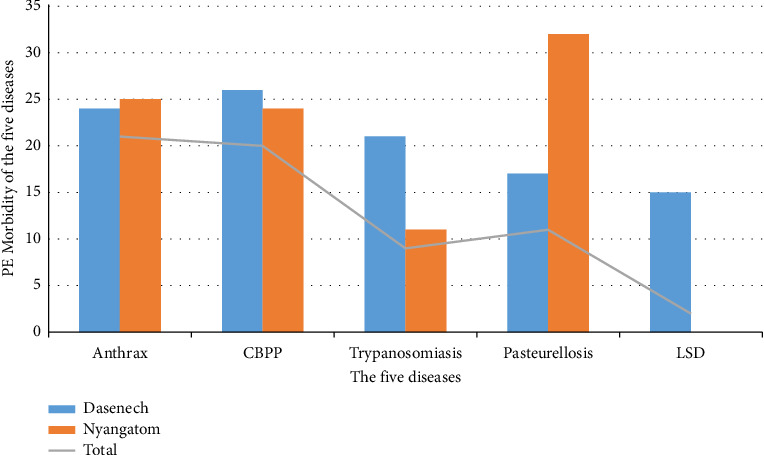
Informants' perception of the morbidity of the five diseases.

According to the informant's perception as determined through the proportional pilling exercise, anthrax, CBPP, trypanosomiasis, pasteurellosis, and LSD were the most commonly observed morbid diseases in the study areas, with the relative prevalence rate (PE-morbidity) of 18%, 16.5%, 6%, 8.5%, and 3.4%, respectively (Figure 6).

#### 3.5.1. Disease Occurrence in Specific Age Groups

**Figure 7:**
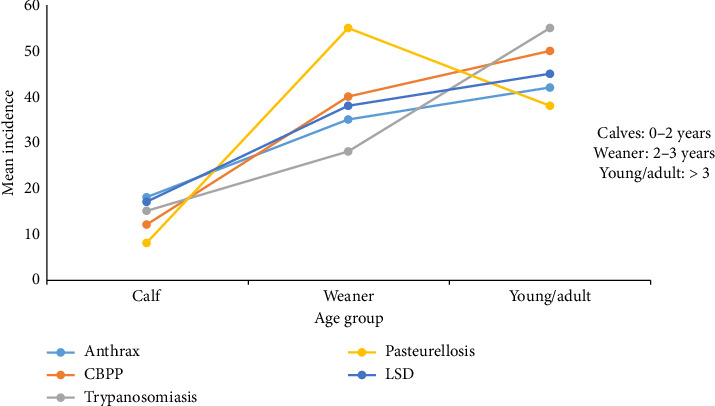
The mean incidence of important diseases in different age groups in both districts.

The pastoralists from selected study kebeles of both districts categorized cattle in the same age groups but named them differently. The Dasenech community categorizes cattle into three age groups: calves, *Made (*Female*)/Agnyap* (Male); Weaner, *Aar*; and Young, *Yer* [15]. The Gnyangatom pastoralists also categorize cattle into three age groups: calves, *Etak*; weaners, *Atak (Female)/Ezeriyot* (Male); and Young, *Ayteng (*Female*)/Emog* (Male),

The disease incidence in specific age groups, as estimated by the pastoralists in the Dasenech and Gnyangatom districts, is illustrated in Figure 7.

According to the result of the proportional piling exercise done by pastoralists in Dasenech and Gnyangatom districts, all the top five diseases had the highest incidence in young/weaner age and lowest in calves. The findings of this study indicated that, though all age groups are susceptible, the incidence of these diseases increases as age increases (young cattle were more susceptible to infection than calves).

### 3.6. Case Fatality of the Five Diseases

**Figure 8:**
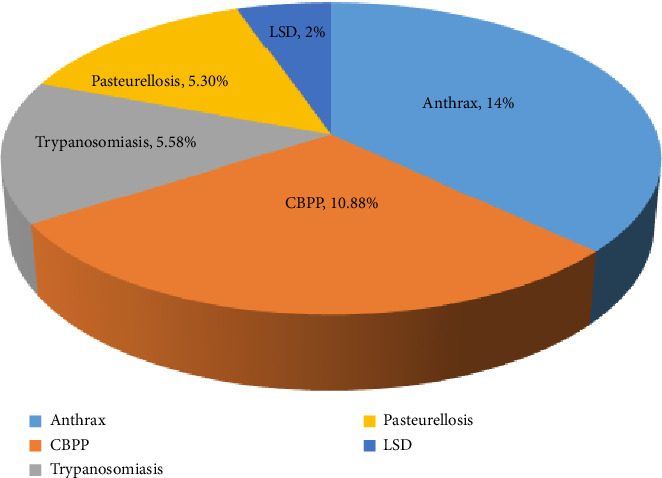
Case fatality rate of the five most prevalent diseases relative to the recovered cattle.

The results of the proportional piling exercise on the case fatality rate showed that 37.76% of cattle died from five important diseases in the past 1 year (Figure 8); of this, anthrax, CBPP, trypanosomiasis, pasteurellosis, and LSD accounted for 14%, 10.88%, 5.58%, 5.3%, and 2% of fatality rates, respectively.

### 3.7. Matrix Scoring

The results of pairwise ranking were the basis for further characterization and selection of diseases for matrix scoring. The analysis of matrix scoring indicated that pastoralists in the study districts have the ability to diagnose cattle diseases, and they have the ability to associate the diseases with their clinical signs (Table 5).

## 4. Discussion

This study revealed that there was almost no variation in the perception of the informants across the districts on the rank of livestock species based on the estimated number and economic importance. The ranking of livestock species in the estimated number and economic value is indicated in Table 1; cattle were highly important for the livelihood of the pastoral people in the study area. This means any development strategies of the agricultural system in the study area should address the health problems of cattle. The cattle population was the highest in number among other livestock species in the study areas, which might be due to pastoralists being heavily dependent on dairy and cattle products as a livelihood system that provides for basic needs. The importance of cattle in these areas surpasses the mere fact of meeting basic needs, since they are traditionally seen as the basis of life, wealth, and social respect. Cattle are the source of food (milk and meat), cash income, and are kept as a capital asset. This result was in agreement with the study results from the Lalibela district [16]. However, the result was in disagreement with Gari et al.'s [17] finding in the Afar region, in which the highest population size was ranked goats, sheep, cattle, camels, donkeys, and chicken, and the priority ranking on the economic value from the highest to the least was goats, camels, cattle, sheep, donkeys, and chicken. Small ruminants made by far the greatest contribution to livestock-based livelihoods in all Afar pastoral communities [18] The arguments to rank goats as a top priority in the Afar region might be due to the fact that goats can resist drought more than cattle, can give birth two times in a year, and they are a quick source of money for the family expenditure. Sheep, cattle, goats, chickens, and donkeys were identified as the common livestock species kept among Maasai pastoralists in Kenya [19], which were also different from the present study result. Reasons given for the higher preference for sheep in the area might include better drought tolerance, reproducing frequently, and steady production of milk. Other studies reported that sheep and goats had a higher advantage than other livestock in the Sekota and Ziquala districts [16]. This was probably due to these districts being semiarid with a harsh environment in which this species was expected to be found predominantly than other livestock species.

The present study also indicated that the vernacular name and the informant's perception of the clinical signs of the same diseases were slightly varied from one district to another (Table 2). The variation of vernacular names between districts may be due to differences in language. The reason behind the difference in clinical signs for the same disease was probably the difference in the level of understanding and in-depth observation of signs by the community. Some diseases were reported all across the districts, but few diseases were specific to one district(s). These might also be due to the informant's ability to reminisce during the SSI.

According to the informant's ranking in the current study (Table 3), it showed that the five diseases in the Dasenech district were anthrax, CBPP, trypanosomiasis, pasteurellosis, and LSD; and CBPP, anthrax, trypanosomiasis, pasteurellosis, and FMD were in the Gnyangatom district. Having the overall median score of the proportional piling exercise in the present study districts, the top five diseases were anthrax, CBPP, trypanosomiasis, pasteurellosis, and LSD (Table 3). Except for fascioliasis and tick infestation, all other diseases were mentioned in the ranking activity of the informant group in both districts. These variations might be due to differences in disease epidemiology because of the unrestricted movement of animals across borders, a common pastoralist way of life, which was one of the predisposing factors that had a great role in the maintenance of arthropod vectors and transmission of the virus/bacteria. Additionally, sharing of the same grazing and watering, differences in kebele selection, differences in informants' scope of knowledge, and remembrance might contribute to the different spread of the disease in the study areas. CBPP, FMD, LSD, blackleg, and mastitis were the five common cattle diseases prioritized by Borana pastoralists [20]. Bahiru and Assefa [16] reported ectoparasites, CBPP, FMD, blackleg, and bloody diarrhea as the major diseases affecting cattle production in Lalibela, Sekota, and Ziquala districts of the Amhara region. According to the findings of Bereket et al. [21], the major five diseases of cattle identified and prioritized in the Dasenech district were CBPP, septicemic pasteurellosis, anthrax, FMD, and blackleg; and in the Hammer district were septicemic pasteurellosis, trypanosomiasis, blackleg, CBPP, and FMD. Maasai pastoralists of Kenya identified malignant catarrhal fever (MCF), East Coast fever (ECF), FMD, CBPP, and AAT as the five most prevalent diseases that affected cattle in their area [19]. Cattle diseases, according to their importance in Pakistan, were mastitis, FMD, internal parasites, and hemorrhagic septicemia, which were ranked as major bovine health issues [22]. The findings of Usman et al. [23] revealed that the prominent diseases affecting cattle in Nigeria were FMD, CBPP, trypanosomiasis, fasciolosis, and black disease. This variation might be due to differences in disease distribution as a result of agroclimatic effect and low treatment success rate. It might also be due to the unrestricted movement of animals across borders. The variation might also be due to the low importance or absence of the disease in their study area.

The findings of this study indicated that disease incidence increased with age, as young cattle were more frequently affected than calves (Figure 7). This trend may be attributed to traditional management practices. In many pastoral settings, calves are often segregated from the main herd, receive closer care, and are less exposed to communal grazing and watering points, which reduces their contact with potential sources of infection. In contrast, young and adult cattle are allowed to move freely, often mix with animals from other herds during migration or at shared resources (unrestricted movement across borders, mixing with new animals, and sharing the same grazing and watering points), increasing their exposure risk. These management-related factors are likely contributors to the observed variation in disease susceptibility among age groups. This finding was in agreement with other studies [20, 24–26], which stated that adult animals have higher odds of being affected compared to calves. In contrast, some studies have reported higher disease incidence among calves than older cattle [27–29]. This may be attributed to suboptimal calf management practices, such as poor housing, overcrowded or unhygienic calf shelters, insufficient colostrum intake, and limited access to veterinary care. In systems where calves are not effectively segregated or monitored, their risk of exposure to pathogens can be higher than that of older, more mobile animals. According to Bahiru and Assefa [16], newborns are mostly sensitive to infectious diseases due to a weak immunity level.

In this study, informants observed that although not all diseases led to high immediate mortality, the economic impact remained substantial, primarily due to weight loss, reduced milk production, and lower market value among animals that survived the infection. According to the proportional piling exercise, the combined case fatality rate for the five most prevalent diseases was 37.76% (Figure 8). Informants were well aware of the economic consequences, not only due to animal loss but also due to the lasting effects of disease on surviving animals' productivity. This highlights the value of integrating pastoralist knowledge into disease surveillance and control strategies, especially in areas where quantitative data may be limited. According to some informants, recovery from severe infection was slow due to emaciation and/or secondary infections. Therefore, it was fair to say that most of the participants in the study area had a good perception and could differentiate and explain the common clinical signs of the five diseases, which fits the clinical observation.

## 5. Conclusion and Recommendations

This study highlights the limited use of participatory methods in pastoral areas and questions their effectiveness while demonstrating the value of PE approaches in gathering crucial information about cattle diseases and assessing community knowledge. This study also highlights a critical gap between pastoralists' indigenous knowledge of livestock diseases and the formal veterinary services that design and implement disease control strategies. While pastoral communities possess deep, experience-based insights into disease patterns, transmission, and local responses, these are rarely integrated into official planning and decision-making processes. It reveals that pastoralists possess significant knowledge about endemic diseases, with anthrax, CBPP, trypanosomiasis, pasteurellosis, and LSD being the most impactful on their livelihoods. The study also provides specific morbidity and case fatality rates for these diseases, with 18%, 16.5%, 6%, 8.5%, and 3.4% PE-morbidity and 14%, 10.88%, 5.58%, 5.3%, and 2% of PE-case fatality, respectively. Despite the wealth of veterinary knowledge among pastoralists, there is a need for greater engagement in sharing their indigenous knowledge on cattle diseases, which can be facilitated through participatory appraisal methods that aid in disease control and contribute to national and regional surveillance programs. Therefore, based on the above conclusive remark, recommendations include, since the absence of structured dialogue and collaboration undermines the effectiveness, relevance, and adoption of control measures, bridging this gap through participatory approaches and inclusive policy-making is essential for sustainable and community-driven livestock health management in pastoral areas by using pastoralists' experiences as a base for planning surveillance, control, and prevention strategies, providing training for veterinary professionals on PE to rule out the problem of livestock sectors, incorporating the identified diseases into control interventions, and conducting further conventional epidemiological studies to address research gaps that are overlooked by the present study.

## Figures and Tables

**Table 1:** Types and rank of livestock based on population and importance.

No	Livestock species	Dasenech	Gnyangatom	Mode^∗^
*Livestock ranked based on the estimated number* ^∗^				
1	Cattle	1	1	1
2	Sheep	4	3	3
3	Goat	2	2	2
4	Donkey	3	5	5
5	Chicken	5	4	4

*Livestock ranked based on economic importance* ^∗^				
1	Cattle	1	1	1
2	Sheep	3	3	3
3	Goat	2	2	2
4	Donkey	4	4	4
5	Chicken	5	5	5

*Note:* ^∗^interpretation: 1 = ranked first, 2 = ranked second, and 5 = ranked fifth.

**Table 2:** Summarized cattle diseases and their vernacular name and clinical descriptions declared by informants.

No	Vernacular name	Scientific name	Clinical manifestations	District(s)
1	Qemudch (ቀሙድች), akatulan (አካቱላን)	Anthrax	Abdominal pain, blood oozes from the nose, and sudden death	Dasenech, Gnyangatom
2	Loke (ሎኬ), lowuko (ሎዉኮ)	Contagious bovine pleuropneumonia (CBPP)	Nasal discharges, painful, and difficult breathing	Dasenech, Gnyangatom
3	Lesukogn (ለሱኮኝ), lokgerion (ሎግሪዮን)	Trypanosomiasis	Rough, standing hair, inappetence, no regurgitation	Dasenech, Gnyangatom
4	Charo (ጫሮ), lokuwas (ሎኩዋስ), lotayen (ሎታይን)	Pasteurellosis	Nasal discharge, cough, rapid breathing	Dasenech, Gnyangatom
5	Verte (ቨርቴ), lomerie (ሎሜሪ)	Lumpy skin disease (LSD)	Nodules on skin, lacrimation, hair stands, milk reduction, lameness, depression	Dasenech, Gnyangatom
6	Belblam (በልብላም), emara (እማራ)	Brucellosis	Abortion, hygromatous swelling	Dasenech, Gnyangatom
7	Wuluf (ዉሉፍ), lokejen (ሎኬጀን)	Foot and mouth disease (FMD)	Lameness, wounds on hooves and mouth, milk reduction	Dasenech, Gnyangatom
8	Gagaba (ጋጋባ), maleri (ማሌሪ)	Listeriosis	Circling disease, depression, poor appetite	Dasenech, Gnyangatom
9	Bergech (በርጌች), emara/Lotulin/Longorie (እማራ/ሎቱሊን/ሎንጎሪ)	Blackleg	Lameness, crackling swelling on the limb	Dasenech, Gnyangatom
10	Emagne (እማኝ)	Fascioliasis	Weight loss, gait abnormality, abdominal pain, hepatomegaly	Gnyangatom
11	Konche (ቆንጬ)	Tick infestation	Presence of ticks on the head, ears, neck, belly, back, legs, perineum, and tail	Dasenech

**Table 3:** Median scores and range indicating the rank of the diseases.

No.	Proportional piling of 100 counters				
	List of cattle diseases	Overall median	Overall range	Dasenech median	Gnyangatom median
1	Trypanosomiasis	14.5	3–24	13	16
2	CBPP	18.5	17–24	18	19
3	Pasteurellosis	10.0	6–15	7	13
4	Anthrax	28.5	9–40	40	17
5	Lumpy skin disease (LSD)	6.0	4–7	7	5
6	Fascioliasis	5.0	0–13	—	5
7	Foot and mouth disease (FMD)	5.5	2–15	2	9
8	Blackleg	1.0	0–5	2	0
9	Tick infestation	5.0	0–4	5	—
10	Listeriosis	1.0	2–5	2	0
11	Brucellosis	1.0	2–17	2	0

*Note:* dash (—) means that the disease was not mentioned, hence included as zero in proportional piling.

**Table 4:** Relative importance of cattle diseases in the study areas using pairwise ranking.

Cattle diseases	Study districts^∗^		
	Dasenech	Gnyangatom	Mean rank
	Rank of cattle disease importance		
Trypanosomiasis	3	3	5.50
CBPP	2	1	5.50
Pasteurellosis	4	4	4.00
Anthrax	1	2	2.75
Lumpy skin disease	5		1.75
Foot and mouth disease		5	1.50

^∗^ *p*=0.112.

**Table 5:** Matrix scoring of disease indicators in the two districts.

Indicators/signs	Diseases				
	Anthrax	CBPP	Trypanosomiasis	Pasteurellosis	LSD
Weight loss	**••••••••**	**••**	••••••••••	**•**	
	**••••••••**	1.5 (0–9)	10 (6–13)	1 (0–7)	0 (0–2)
	15.5 (9–25)				

Hair erection	**•**	**•**	**••••••••**	**••••**	**•**
	0.5 (0–40)	1 (0–8)	**•••••••**	4 (0–5)	0.5 (0–5)
			15 (10–20)		

Mortality		**•••**	•••••••	**••••••••••**	
	**••••••••**	3 (0–6)	7 (0–11)	10 (8–17)	0 (0–0)
	8 (0–10)				

Respiratory signs	**••**	••••••••••		**••••••••••**	
	1.5 (0–5)	••••••••	0 (0–0)	**••••••••**	0 (0–0)
		15 (13–20)		18 (12–25)	

Milk reduction	**••••••••••**	**•••**	**•••**	**••••••**	**•**
	10 (9–12)	2.5 (0–7)	3 (0–4)	6 (4–10)	0.5 (0–3)

Skin lesion			**•••••••**	**••**	**••••••••**
	0 (0–0)	0 (0–0)	7 (0–11)	1.5 (0–3)	**••••••••**
					15.5 (13–25)

*Note:* the black dots represented the median. The range was shown in parentheses. There was moderate agreement (*W* between 0.26 and 0.38) between informant groups (overall *W* = 0.382; *p*=0.034).

## Data Availability

The data used to support the findings of this study are available at Jinka University, Jinka, Ethiopia.
